# Alcohol Use Trajectories During the First 72 Weeks of WHOOP Wearable Platform Membership: Observational Cohort Study

**DOI:** 10.2196/91288

**Published:** 2026-04-27

**Authors:** Gregory J Grosicki, William von Hippel, Finnbarr Fielding, Christopher J Chapman, David M Presby, Josh Leota, Kristen E Holmes

**Affiliations:** 1 Department of Performance Science and Health Outcomes, WHOOP, Inc., One Kenmore Square, Boston, MA, 02215, United States, 1 703-303-7812; 2 Department of Data Science, WHOOP, Inc., Boston, MA, United States; 3 SIB Swiss Institute of Bioinformatics, Lausanne, Vaud, Switzerland; 4 Department of Computational Biology, University of Lausanne, Lausanne, Vaud, Switzerland; 5 Division of Sleep and Circadian Disorders, Department of Medicine, Brigham and Women's Hospital, Boston, MA, United States; 6 School of Psychological Sciences, Monash University, Victoria, Australia

**Keywords:** behavior change, digital health, alcohol consumption, wearable technology, real-world data, health behavior, self-monitoring

## Abstract

Among 30,000 new wearable device users (11.6 million person-days), self-reported alcohol consumption significantly declined over 72 weeks, with the daily probability of drinking decreasing by 5.8 percentage points (*P*<.001) and reductions across age and biological sex.

## Introduction

Alcohol use is a major contributor to global morbidity and mortality [1], and even low levels of consumption confer measurable health risk [2]. Despite growing awareness of this risk, alcohol remains among the most widely used drugs worldwide [3]. Although behavioral interventions can reduce intake, effects are often modest and short-lived [4], highlighting the need for scalable strategies supporting sustained, population-level reductions in alcohol use.

Wearable devices have become increasingly popular for continuous health monitoring, enabling real-time feedback on physiology and behavior. However, evidence linking wearable use to alcohol-related behaviors remains limited. A recent randomized controlled trial found that a wearable-supported behavioral program was associated with clinically meaningful reductions in alcohol use over 12 weeks [5]. To evaluate whether similar patterns emerge at scale and persist over longer durations, we examined longitudinal trajectories of self-reported alcohol consumption during the first 72 weeks of wearable membership in a large, real-world cohort.

## Methods

### Study Population

We studied 30,000 international adults (50.0% females) aged 18‐79 years who joined the WHOOP platform (WHOOP, Inc.) in 2023. Participants enrolled throughout the year and contributed up to 72 weeks of data, structured into consecutive seven-day weeks anchored to each participant’s join date. Quarters (Q1−Q6) were defined as consecutive 12-week intervals. Eligibility required at least 18 seven-day weeks with complete alcohol use reporting (≥25% of the observation window) [6], with additional weeks containing ≥3 entries also retained (Multimedia Appendix 1). Stratified random sampling by sex ensured balanced representation.

### Self-Reported Alcohol Use

Daily alcohol use was self-reported through the WHOOP smartphone application via a customizable morning journal prompt asking users to confirm behaviors from the prior day (Figure S1 in Multimedia Appendix 1). Users indicated whether alcohol was consumed and could optionally report the number of drinks. Alcohol logging was not required for device use. Only days marked explicitly as “yes” or “no” were analyzed.

Members could also set personalized alcohol-related goals within the application, which could range from abstaining entirely to limiting drinking on specific days. Due to heterogeneity in goal types, goal setting was analyzed as a binary indicator (any goal vs none).

### Statistical Analysis

Drinking frequency was modeled as the daily probability of alcohol use within each week using generalized linear mixed-effects models (binomial, logit link) with fixed effects for quarter, age, sex, season, total weeks contributed, and weekend proportion, with participant-level random intercepts. Estimated marginal means were computed by quarter and compared with Q1 using Dunnett-adjusted contrasts. Sensitivity analyses and modeling details are described in the Multimedia Appendix 1. All analyses were performed in R (version 4.2.2; R Foundation for Statistical Computing) with statistical significance set at *α*=.05.

### Ethical Considerations

Participants consented to their anonymized data being used for research purposes. The study protocol was approved by an independent institutional review board (Salus IRB #251121, Austin, TX). Participants did not receive compensation for their participation in this study.

## Results

The final dataset included 11,651,827 person-days (1,776,673 person-wk). Mean age was 34.4 (SD 10.5) yrs, BMI was 25.1 (SD 4.0) kg/m^2^, and participants contributed a mean of 59.2 (SD 13.5) weeks (median 65 wk, IQR 18‐72 weeks), with an average of 6.6 (SD 0.9) alcohol entries per week (Table S1 in Multimedia Appendix 1).

**Figure 1.**
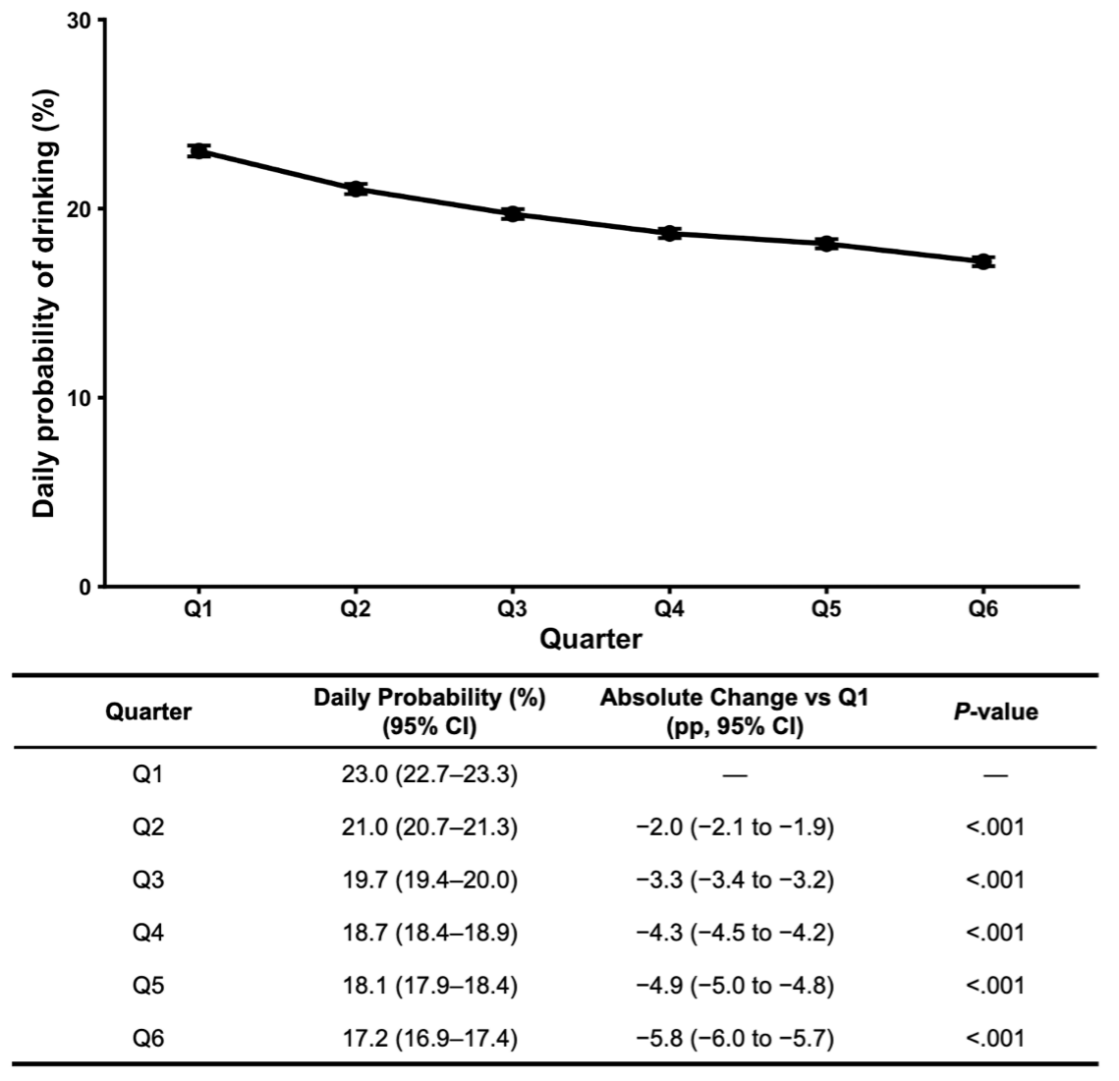
Adjusted daily probability of alcohol use during the first 72 weeks of membership. Marginal estimates from a binomial generalized linear mixed-effects model are shown for six sequential 12-week quarters (Q1–Q6). Points represent model-based predicted probabilities that a logged day was a drinking day, and error bars indicate 95% CIs. Absolute changes versus Q1 (percentage points) and corresponding Dunnett-adjusted *P* values are derived from model-based contrasts. The model included fixed effects for quarter, age, biological sex, season, total number of weeks contributed, and the proportion of days in each user-week that fell on a weekend, with a random intercept for participant.

The daily probability of alcohol use declined over time (Figure 1), from 23.0% (95% CI 22.7 to 23.3) in Q1 to 17.2% (95% CI 16.9 to 17.4) in Q6, an absolute reduction of 5.8 percentage points (95% CI −6.0 to −5.7). Results were robust in sensitivity analyses, including restriction to complete seven-day drink number reporting, where drinking volume decreased by 1.11 drinks per week (95% CI −1.15 to −1.07; Figure S2 in Multimedia Appendix 1).

**Figure 2.**
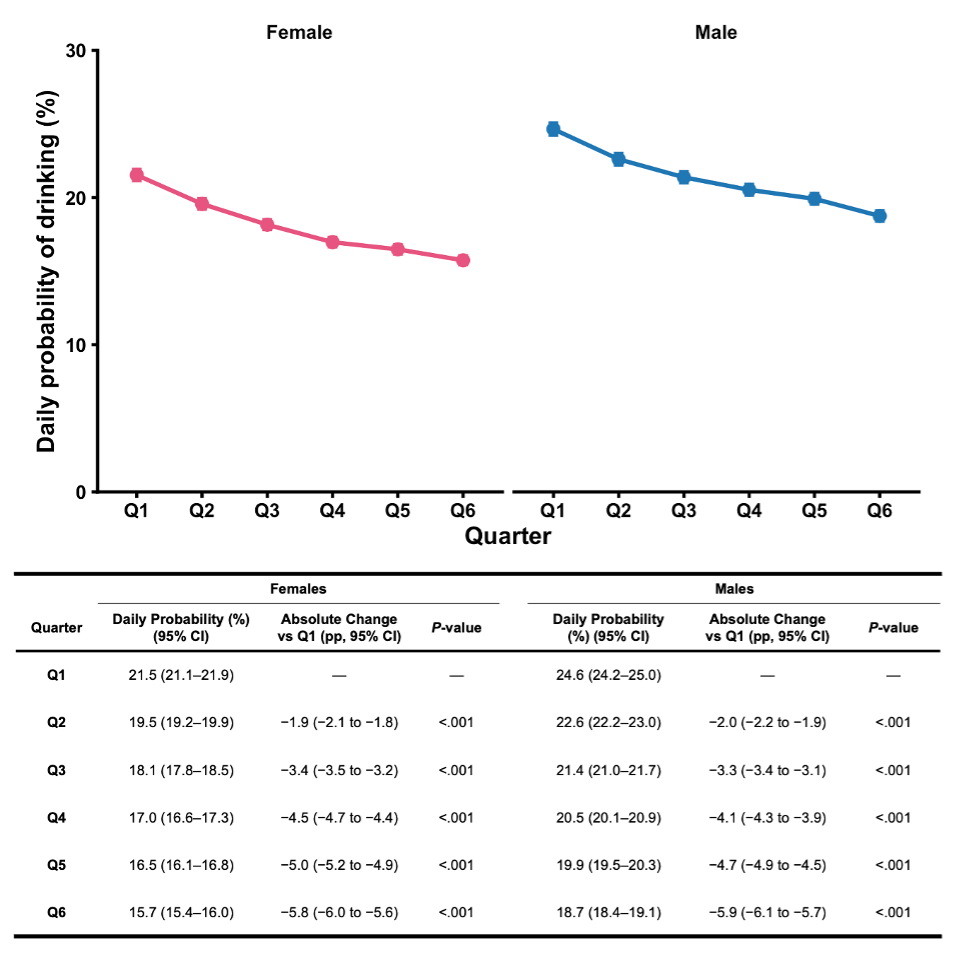
Adjusted daily probability of alcohol use during the first 72 weeks of membership, stratified by biological sex. Estimated marginal means from a binomial generalized linear mixed-effects model are shown separately for females and males across six sequential 12-week quarters (Q1–Q6). Points represent model-based predicted probabilities that a logged day was a drinking day, and error bars indicate 95% CIs. Absolute percentage-point changes relative to Q1 and associated Dunnett-adjusted *P* values were derived from model-based contrasts within each sex. Models adjusted for age, season, total number of weeks contributed, and the proportion of days in each user-week that fell on a weekend, with a participant-level random intercept.

**Figure 3.**
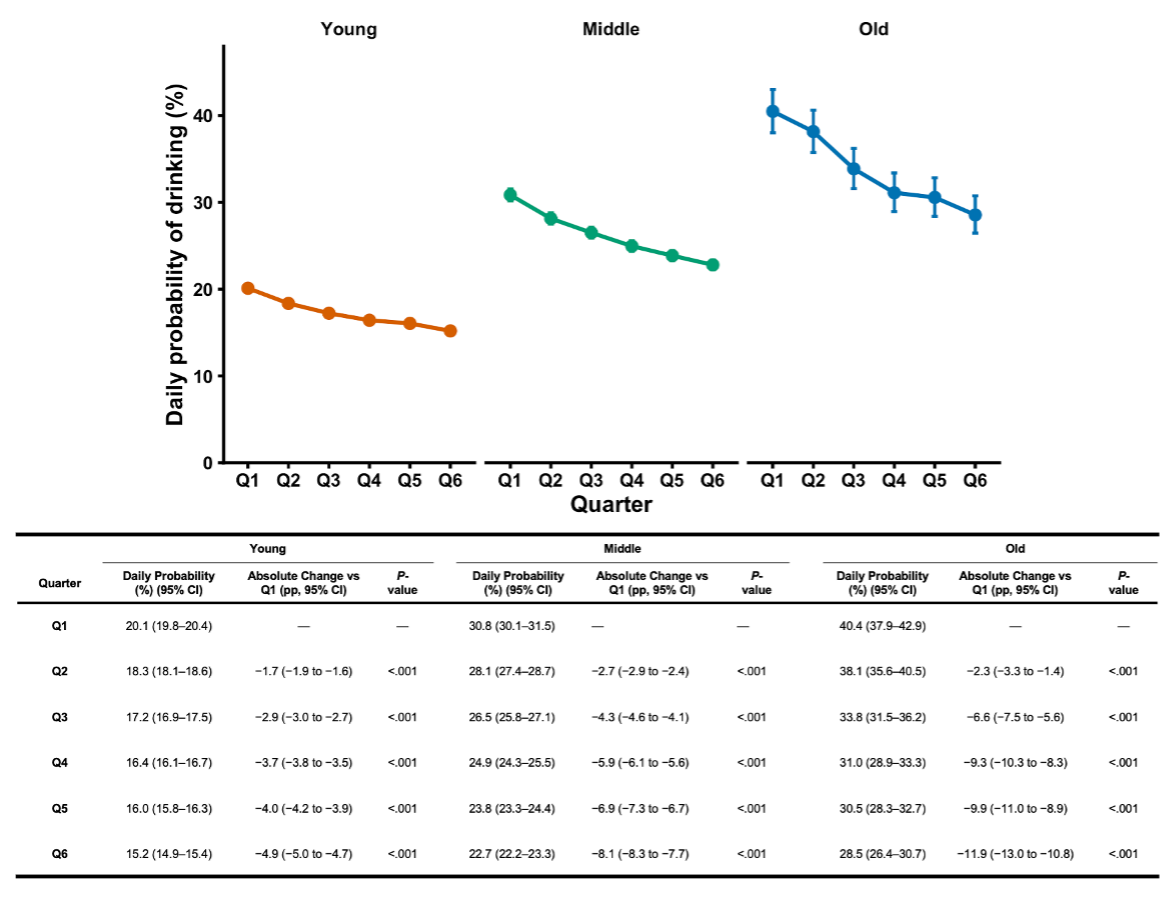
Adjusted daily probability of alcohol use during the first 72 weeks of membership, stratified by age group. Estimated marginal means from a binomial generalized linear mixed-effects model are shown separately for participants aged 18–39 years (n = 21,670), 40–59 years (n = 7,610), and 60–79 years (n = 720) across six sequential 12-week quarters (Q1–Q6). Points represent model-based predicted probabilities that a logged day was a drinking day, and error bars indicate 95% CIs. Absolute percentage-point changes relative to Q1 and corresponding Dunnett-adjusted *P* values were derived from model-based contrasts within each age group. Models adjusted for biological sex, season, total number of weeks contributed, and the proportion of days in each user-week that fell on a weekend, with a participant-level random intercept.

The baseline daily drinking probability was higher among males than females (*P*<.001), but the relative decline from Q1 to Q6 was greater in females (*P*<.001; Figure 2). Baseline daily drinking probability differed across age groups (*P*s <.001). Progressively larger relative and absolute declines in drinking were observed with increasing age (*P*s <.001; Figure 3).

Both goal setters (n=13,298; Table S2 in Multimedia Appendix 1) and nongoal setters showed significant declines, though the relative decline was greater among non-goal setters (*P*<.001; Figure S5 in Multimedia Appendix 1).

## Discussion

In this large real-world cohort of 30,000 WHOOP members, self-reported alcohol use declined over the first 72 weeks of membership, with both drinking frequency and volume decreasing across age and sex. These findings extend recent experimental results [5] by describing sustained reductions in alcohol use over 72 weeks in a large real-world cohort.

The observed declines may reflect increased awareness of health behaviors, heightened sensitivity to physiological feedback, or broader lifestyle changes after wearable enrollment [7]. Importantly, participants engaged in active self-monitoring through journal entries, a behavior shown to promote reductions in drinking [8]. Additionally, large-scale wearable-based analyses demonstrate that even moderate alcohol consumption disrupts cardiac autonomic regulation, sleep, and next-day physical activity [9]. Awareness of these consequences may reinforce behavior change. Notably, declines in drinking frequency were accompanied by reductions in volume, suggesting decreased frequency was not offset by increased intake on drinking occasions. Although causal effects cannot be inferred, the persistence of these changes over more than one year highlights the potential for digital health platforms to support sustained alcohol-related behavior change at scale.

Several limitations should be considered. Alcohol use was self-reported without standardized drink definitions, and participants were subscription-based wearable users who may be more health-conscious than the general population. The requirement for sustained reporting engagement (≥18 wk) may further limit generalizability. Although reporting frequency remained relatively high across quarters and sensitivity analyses restricted to complete reporting weeks and members with full 72-week follow-up yielded nearly identical results, informative missingness cannot be excluded. Without a control group, observed changes cannot be causally attributed to wearable enrollment or distinguished from secular trends [10]. Although statistically significant, absolute changes were modest, and clinical significance at the individual level cannot be determined. However, dose-response evidence indicates that alcohol-related health risks increase continuously with consumption [11], with no established safe threshold [12], suggesting even modest population-level reductions may carry public health relevance, particularly when sustained over time and observed across demographic subgroups. Longer follow-up is needed to evaluate persistence beyond the study window. Despite these limitations, this study provides one of the largest longitudinal assessments of self-reported alcohol use to date, describing long-term trajectories of drinking frequency and volume over 72 weeks among new WHOOP members.

## Supplementary material

10.2196/91288 Multimedia Appendix 1 Extended methods, sensitivity analyses, and subgroup results with supporting tables and figures.

## Abbreviations

IRB: institutional review board
Q1–Q6: Quarter 1 through Quarter 6
