# Supplementary material for: Alcohol Use Trajectories During the First 72 Weeks of WHOOP Wearable Platform Membership: Observational Cohort Study
Source: JMIR Mhealth Uhealth. 2026 Apr 27;14:e91288. doi: 10.2196/91288 (PMC13119388; doi:10.2196/91288)
Supplement: Multimedia Appendix 1 [file mhealth-v14-e91288-s001.docx]

**Multimedia Appendix 1**

Extended methods, sensitivity analyses, and subgroup results with supporting tables and figures.

**Supplemental methods**

*WHOOP platform description*

WHOOP is a subscription-based wearable platform consisting of a wrist-worn device and companion smartphone application (**Figure S1**). WHOOP collects physiological signals, including heart rate (via photoplethysmography) and accelerometry (via 3-axis accelerometer), which are used to derive sleep, recovery, and strain metrics displayed in the smartphone application. Members may optionally complete a customizable daily journal reporting health behaviors, including alcohol use, which are integrated with physiological and sleep metrics. Data are aggregated within the WHOOP ecosystem for personalized insights and recommendations.

*Detailed participant eligibility criteria*

Eligibility required at least 18 seven-day weeks with complete alcohol use reporting (≥25% of the 72-week observation window), a threshold consistent with adherence benchmarks in mobile health self-monitoring [6], defined as seven explicit “yes” or “no” entries during the week. These weeks did not need to be consecutive and could occur at any time during follow-up. Beyond these complete weeks, weeks with at least three alcohol journal entries were also included to capture meaningful reporting even if all seven days were not explicitly logged. This threshold was selected a priori to balance adequate longitudinal coverage with retention of a broadly representative cohort rather than restricting analyses to only highly consistent reporters. Participants were not recruited into a separate study. All members consented to the use of their de-identified data for research purposes at account creation under WHOOP’s Terms of Service.

*Detailed statistical analysis plan*

Weekly outcomes were specified as the number of drinking and non-drinking days, allowing the binomial denominator to vary according to the number of completed alcohol journal entries (range: 3−7 days). Fixed effects included prespecified 12-week quarters (Q1−Q6), age, biological sex, season, total weeks contributed, and the proportion of days in each user-week that fell on a weekend (Saturday or Sunday entries, which reflect Friday and Saturday night drinking), with participant-level random intercepts. Models including random slopes for quarter were explored but did not converge. Models were first fit in the overall sample, followed by evaluation of effect modification by biological sex and age group (young=18−39 yrs, n=21,670; middle=40−59 yrs, n=7,610; old=60−79 yrs, n=720). Estimated marginal means were computed on the response scale to obtain model-predicted daily drinking probabilities by quarter, and each quarter was compared with Q1 using Dunnett-adjusted contrasts. Sensitivity analyses were conducted among members contributing at least one week with complete seven-day drink-number reporting. Weekly drink totals were modeled using negative binomial mixed-effects models to account for overdispersion in count data. Additional sensitivity analyses compared trajectories between members who set an alcohol-related goal in-app during follow-up and those who did not.

**Supplemental results**

*Person-week definition*

Person-weeks were defined as consecutive seven-day intervals beginning on each participant’s join date and were retained if they contained ≥3 explicit “yes” or “no” alcohol entries. Accordingly, person-weeks do not necessarily represent seven fully observed person days.

*Reporting engagement over time*

Reporting engagement remained relatively high across follow-up, with a mean of 6.7±.8 completed alcohol entries per week in Q1 and 6.3±1.1 in Q6. The percentage of weeks with all “no” entries varied by reporting week completeness (**Figure S7**).

*Drinking frequency sensitivity analyses*

Estimates were similar in sensitivity analyses restricted to person-weeks with complete seven-day reporting (1,306,681 of 1,776,673 person-weeks; 73.6%), which yielded a 5.7 percentage point reduction (95% CI: −5.9 to −5.6), and when restricted to members with complete 72-week follow-up (n=6,999; 503,928 person-weeks), which also yielded a 5.7 percentage point reduction (95% CI: -6.0 to -5.5).

*Drinking frequency changes by sex and age group*

The decline from Q1 to Q6 differed by sex (β=.04, indicating a steeper relative decline among females on the log-odds scale; *P*<.001). The decline from Q1 to Q6 differed by age group (β=−.07 for middle-aged and β=−.19 for older adults, relative to young adults, on the log-odds scale; *P*s<.001), corresponding to progressively larger absolute declines with increasing age (*Ps*<.001; **Figure 3**). Across all groups, drinking was disproportionately concentrated on weekends, with this pattern most pronounced in young adults (**Figure S8**).

*Drink count sensitivity analyses*

In sensitivity analyses restricted to weeks with complete seven-day drink-number reporting (**Figure S2−S4**), 29,947 members contributed 1,240,374 person-weeks, representing 69.8% of the 1,776,673 total observed person-weeks. Mean total weekly drinks declined from 3.40 (95% CI: 3.34 to 3.46) in Q1 to 2.29 (2.25 to 2.33) in Q6, an absolute reduction of 1.11 drinks per week (95% CI: −1.15 to −1.07). Stratified analyses showed similar patterns by sex (females: −.93 drinks per week [95% CI: −.98 to −.89]; males: −1.32 [95% CI: −1.39 to −1.26]) and age group (young: −1.12 drinks per week [95% CI: −1.18 to −1.07]; middle: −1.10 [95% CI: −1.18, −1.01]; old: −.97 [95% CI: −1.18 to −.75]).

*Goal setting comparison*

Compared with members who did not set goals, goal-setting members were similar in age (34.3±10.8 vs 34.5±10.0 years) and BMI (25.0±4.0 vs 25.1±4.0 kg/m²; *Ps*≥.08), although a greater proportion were female (52.9% vs 47.7%; *P*<.001). Baseline daily drinking probability and mean weekly drink totals were higher among goal setters (*P*<.001). The decline from Q1 to Q6 differed between goal setters and non-goal setters (β=0.04, indicating a steeper relative decline among non-goal setters on the log-odds scale; *P*<.001). Absolute declines were 6.0 percentage points in goal setters (95% CI: −6.2 to −5.7) and 5.8 percentage points in non-goal setters (95% CI: −5.9 to −5.6; **Figure S5**). Comparable patterns were observed when modeling weekly drink totals (**Figure S6**).

| **Table S1.** Participant and reporting engagement across quarters | | | | |
| --- | --- | --- | --- | --- |
| **Quarter** | **Participants** | **Person-Weeks** | **Total Days** | **Days Per Week** |
| **1** | 29,913 | 344,183 | 2,292,292 | 6.7 ± .8 |
| **2** | 29,681 | 341,685 | 2,270,286 | 6.6 ± .8 |
| **3** | 29,416 | 329,977 | 2,176,691 | 6.6 ± .8 |
| **4** | 27,986 | 303,415 | 1,982,334 | 6.5 ± .9 |
| **5** | 25,144 | 252,796 | 1,632,996 | 6.5 ± 1.0 |
| **6** | 20,199 | 204,617 | 1,297,228 | 6.3 ± 1.1 |

| **Table S2.** Distribution of alcohol-related goals among goal setting users | | |
| --- | --- | --- |
| **Alcohol Free Days Goal** | **Users** | **% of Goal Setters (n=13,298)** |
| 1 | 1,125 | 8.5 |
| 2 | 514 | 3.9 |
| 3 | 774 | 5.8 |
| 4 | 7,775 | 58.5 |
| 5 | 4,957 | 37.3 |
| 6 | 2,260 | 17.0 |
| 7 | 4,483 | 33.7 |
| *Total users exceed the number of users who set a goal (n=13,298) because users could set multiple distinct goals over the observation period. | | |


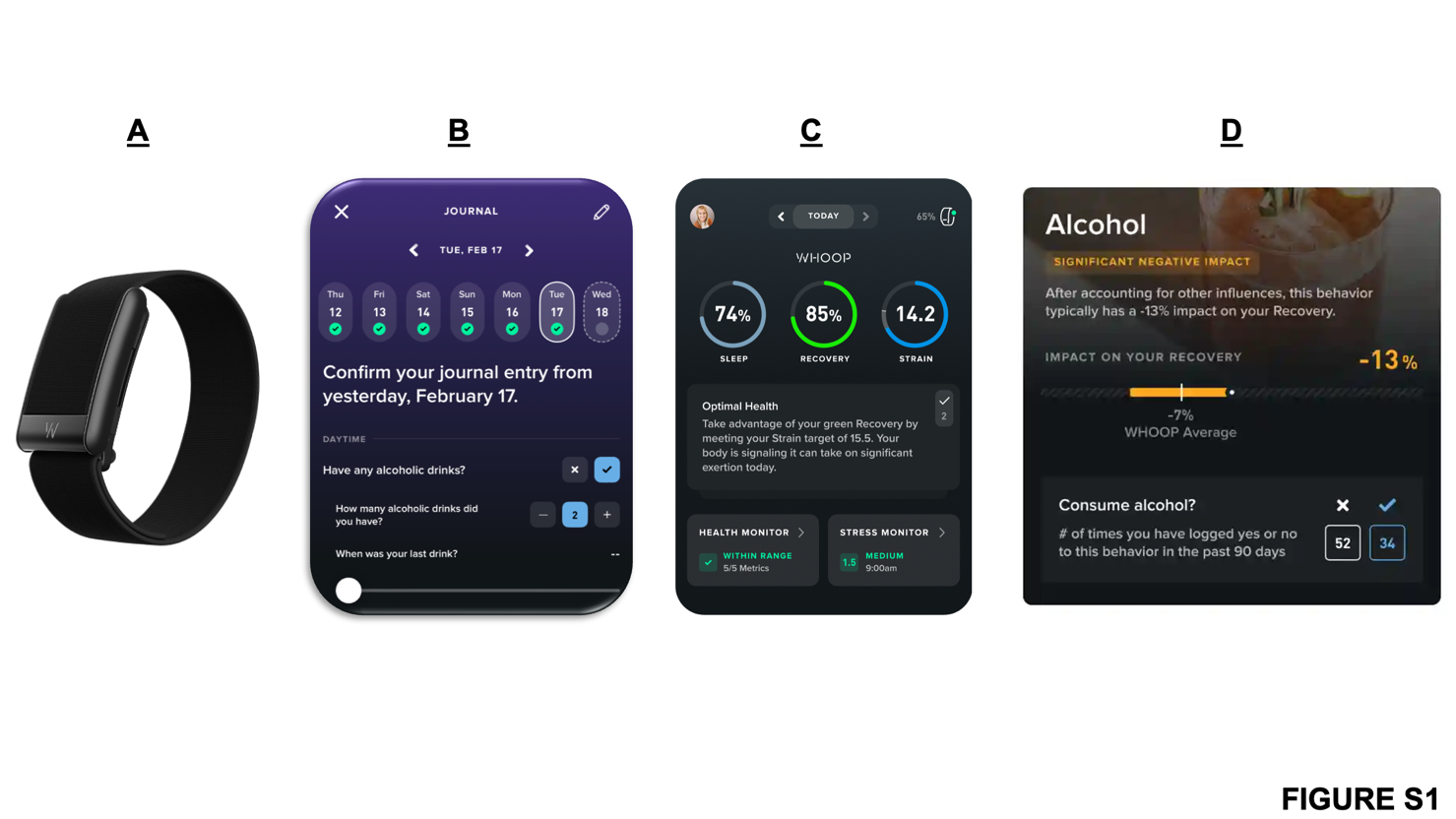


**Figure S1. Overview of the WHOOP wearable device, smartphone application, and alcohol journal workflow.** (**A**) Wrist-worn WHOOP device used to continuously collect physiological signals, including heart rate via photoplethysmography and movement via a 3-axis accelerometer. (**B**) Example of the customizable daily journal interface within the WHOOP smartphone application, where members may self-report prior-day behaviors, including alcohol consumption (yes/no) and optional number of drinks. (**C**) Example of the application dashboard displaying derived metrics such as sleep, recovery, and strain. (**D**) Example of behavioral feedback provided within the application, illustrating the estimated impact of logged alcohol use on recovery metrics.


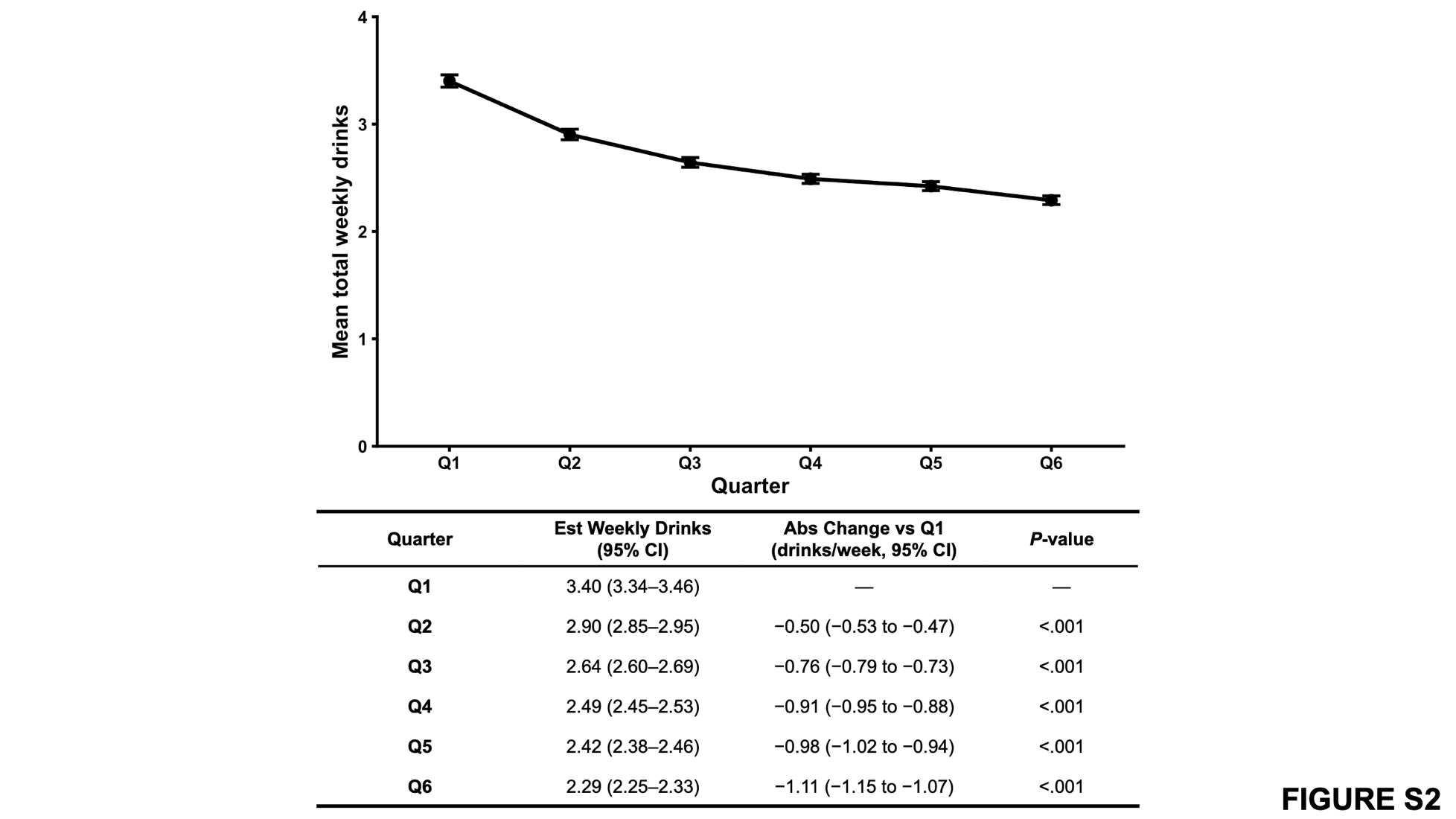


**Figure S2. Longitudinal changes in adjusted mean weekly alcohol consumption during the first 72 weeks of wearable membership.** Marginal estimates from a negative binomial generalized linear mixed-effects model showing the mean number of drinks per week across six sequential 12-week quarters. Points represent model-based predicted means and error bars denote 95% confidence intervals. The model included fixed effects for quarter, age, biological sex, season, and total number of weeks contributed, with a random intercept for participant. Absolute changes in drinks per week relative to Quarter 1 are shown below the figure with corresponding 95% confidence intervals and Dunnett-adjusted p-values.


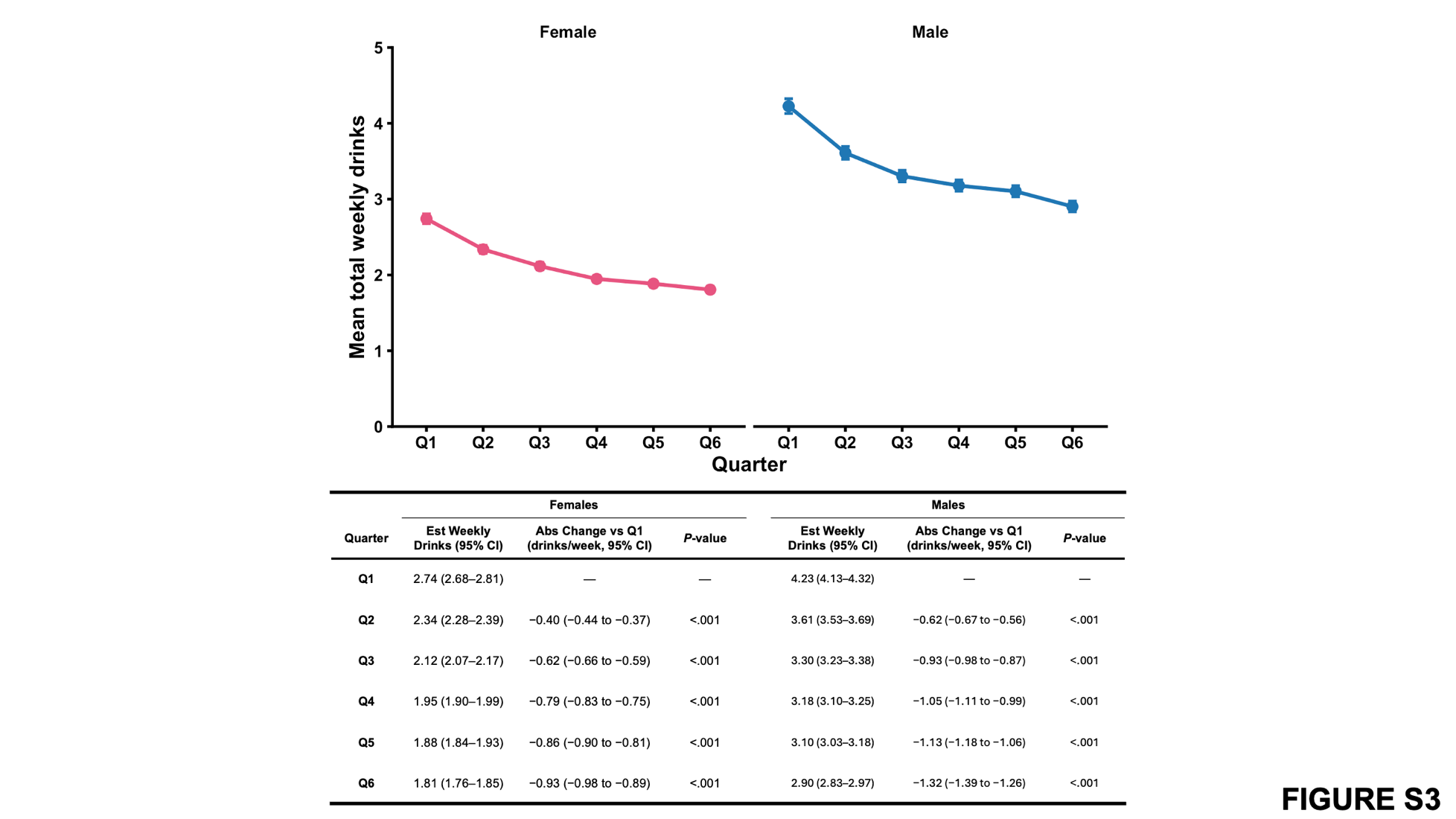


**Figure S3. Sex-stratified changes in adjusted mean weekly alcohol consumption.** Estimated marginal means from negative binomial generalized linear mixed-effects models showing predicted mean weekly drink totals across six sequential 12-week quarters (Q1–Q6), stratified by sex. Points represent model-based predicted means and error bars denote 95% confidence intervals. Models included fixed effects for quarter, age, season, and total number of weeks contributed, with a participant-level random intercept. Absolute changes in drinks per week relative to Quarter 1 are provided with corresponding 95% confidence intervals and Dunnett-adjusted p-values.


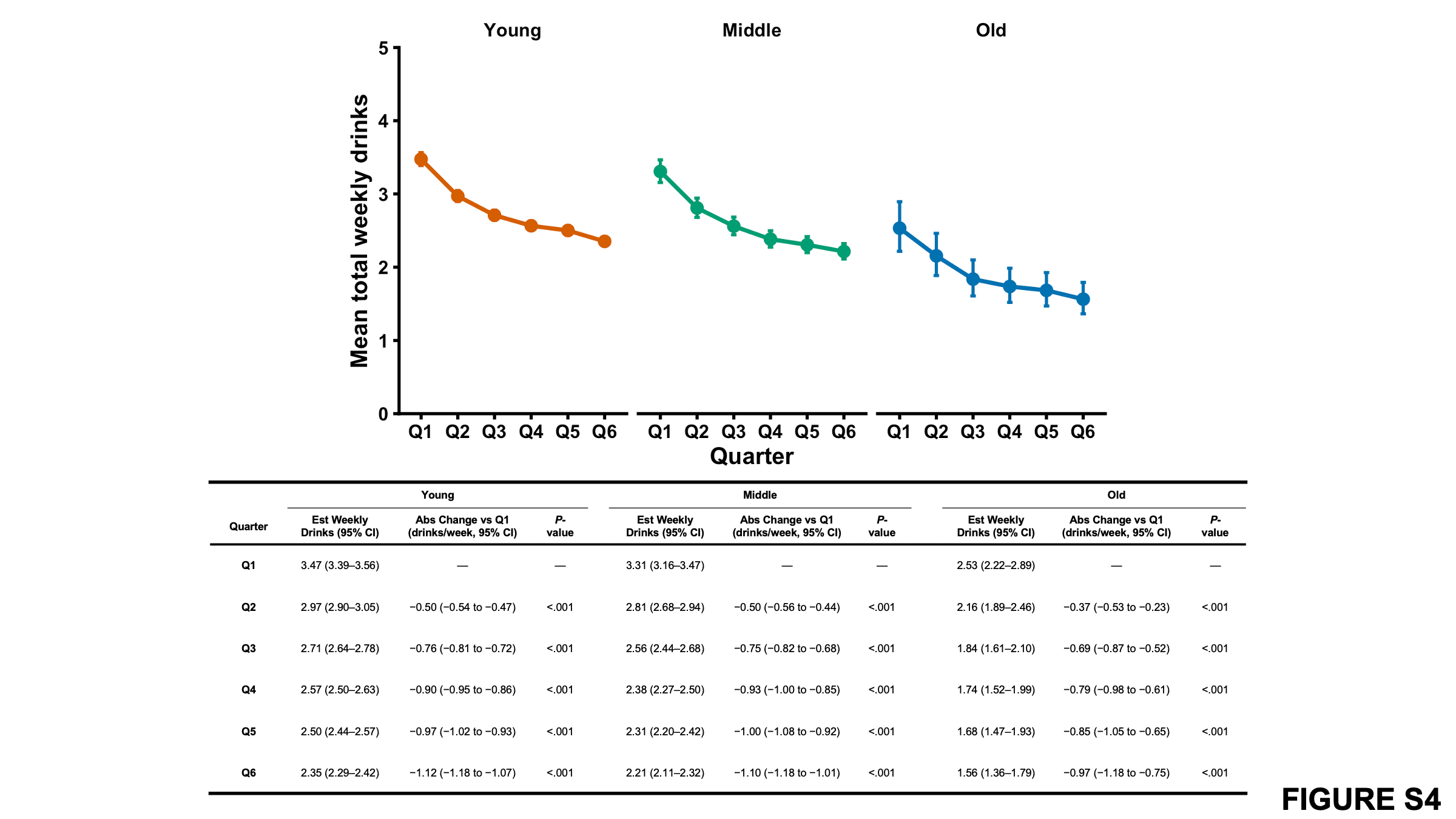


**Figure S4. Age group–stratified changes in adjusted mean weekly alcohol consumption.** Estimated marginal means from negative binomial generalized linear mixed-effects models showing predicted mean weekly drink totals across six sequential 12-week quarters (Q1–Q6), stratified by age group (18–39 years, 40–59 years, 60–79 years). Points represent model-based predicted means and error bars denote 95% confidence intervals. Models included fixed effects for quarter, biological sex, season, and total number of weeks contributed, with a participant-level random intercept. Absolute changes in drinks per week relative to Quarter 1 are provided with corresponding 95% confidence intervals and Dunnett-adjusted p-values.


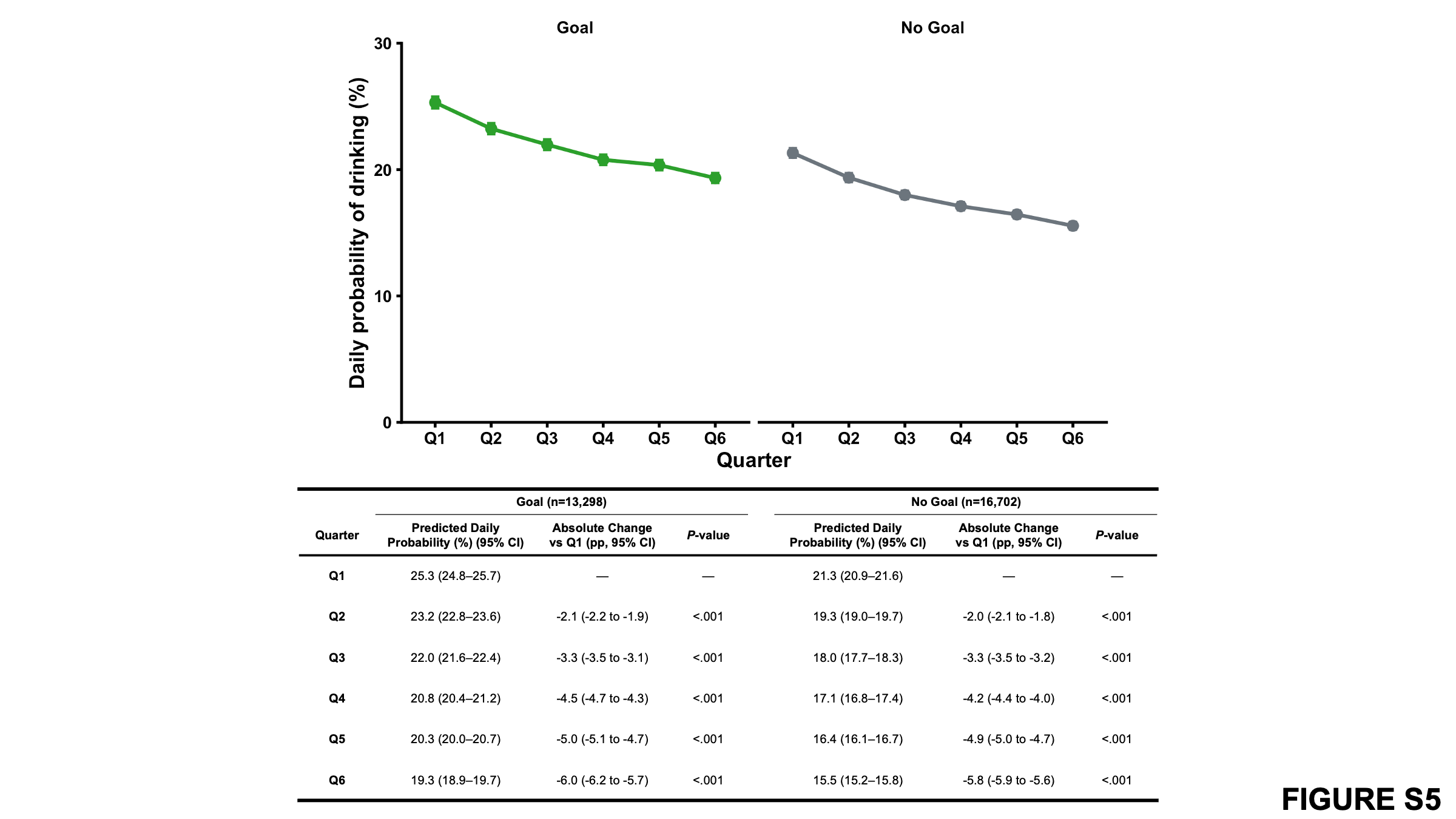


**Figure S5. Adjusted daily probability of alcohol use during the first 72 weeks of membership, stratified by members who made an alcohol-related goal compared to those who did not.** Estimated marginal means from a binomial generalized linear mixed-effects model are shown separately for goal setters and non-goal setters across six sequential 12-week quarters (Q1–Q6). Points represent model-based predicted probabilities that a logged day was a drinking day, and error bars indicate 95% confidence intervals. Absolute percentage-point changes relative to Q1 and associated Dunnett-adjusted p-values were derived from model-based contrasts within each goal-setting group. Models adjusted for age, biological sex, season, total number of weeks contributed, and the proportion of days in each user-week that fell on a weekend, with a participant-level random intercept.


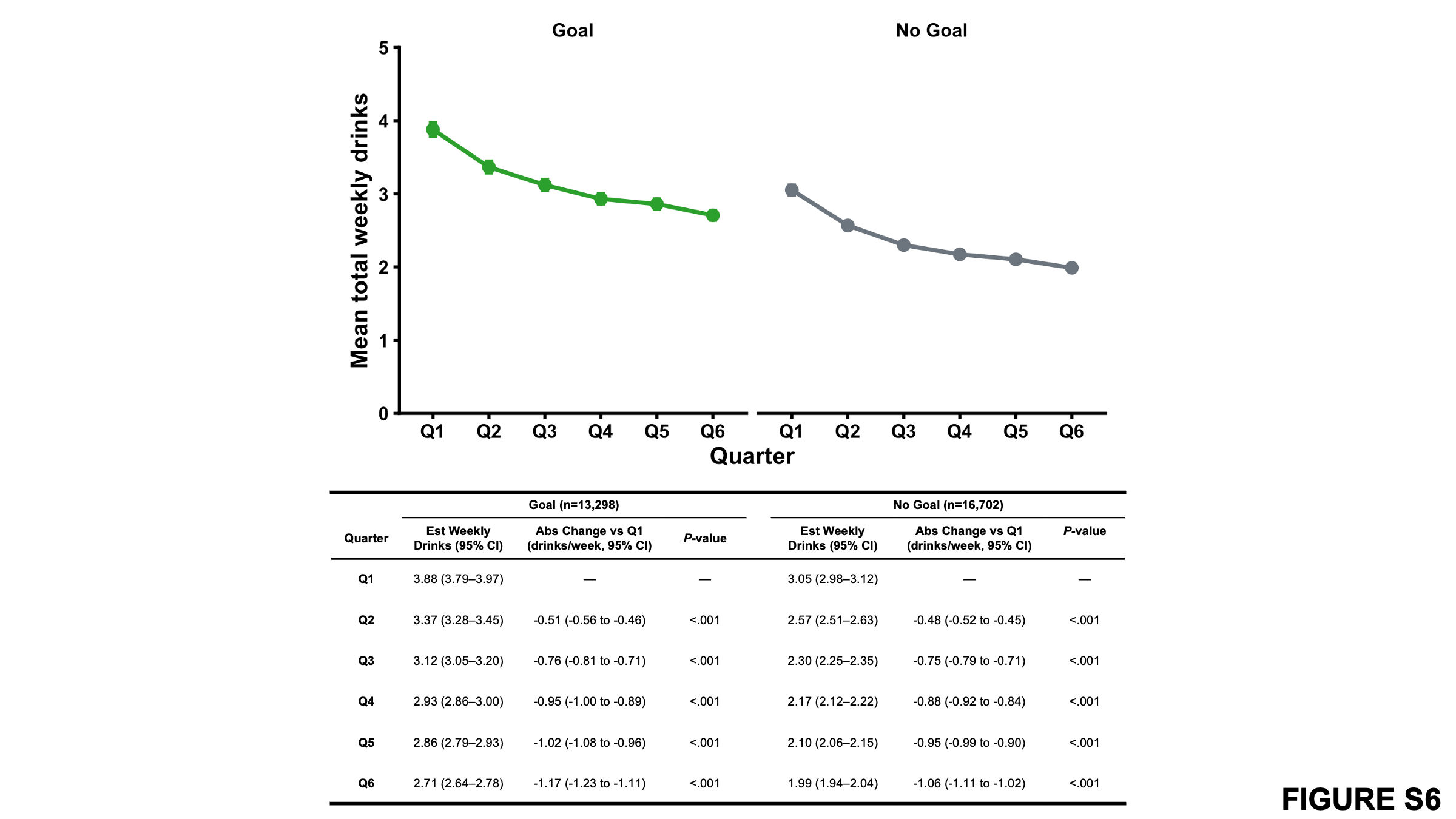


**Figure S6. Goal vs. non-goal stratified changes in adjusted mean weekly alcohol consumption.** Estimated marginal means from negative binomial generalized linear mixed-effects models showing predicted mean weekly drink totals across six sequential 12-week quarters (Q1–Q6), stratified by goal setting. Points represent model-based predicted means and error bars denote 95% confidence intervals. Models included fixed effects for quarter, age at activation, biological sex, season, and total number of weeks contributed, with a participant-level random intercept. Absolute changes in drinks per week relative to Quarter 1 are provided with corresponding 95% confidence intervals and Dunnett-adjusted p-values.


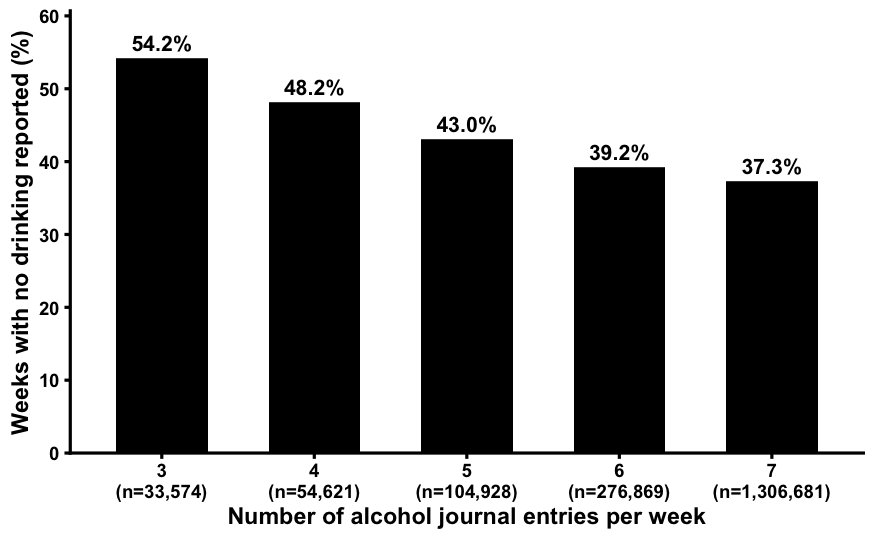


**Figure S7.** Percentage of weeks with no drinking reported (all “no” entries) by number of alcohol journal entries per week. Sample sizes (n) represent the total number of person-weeks at each reporting level.


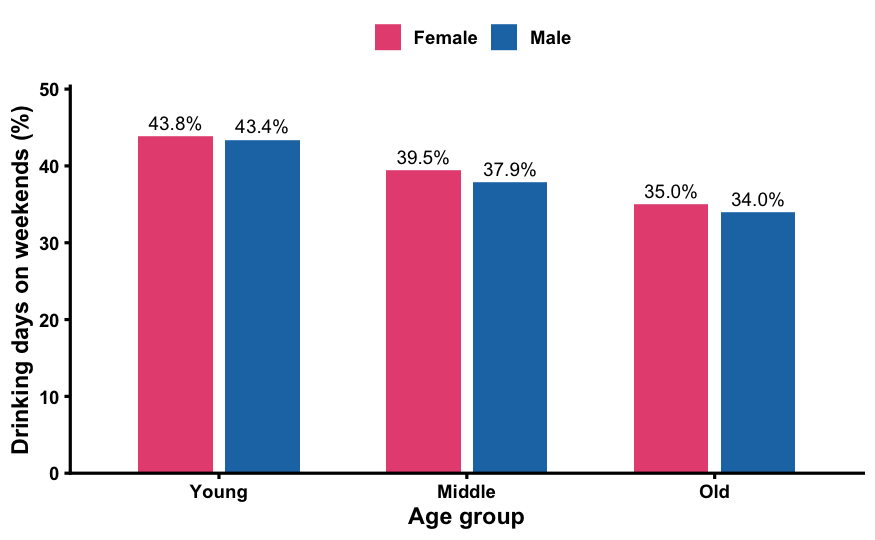


**Figure S8.** Percentage of drinking days occurring on weekends (Saturday-Sunday journal entries, reflecting Friday-Saturday drinking occasions) by age group and biological sex.
